# Digital laser-induced printing of MoS_2_

**DOI:** 10.1515/nanoph-2022-0736

**Published:** 2023-02-28

**Authors:** Adamantia Logotheti, Adi Levi, Doron Naveh, Leonidas Tsetseris, Ioanna Zergioti

**Affiliations:** School of Applied Mathematics and Physical Sciences, National Technical University of Athens – Zografou Campus, Zografou, Greece; Faculty of Engineering, Bar-Ilan University, Ramat Gan, Israel

**Keywords:** digital transfer, laser-assisted transfer, LIFT, moldybdenum disulfide (MoS_2_), monolayer moldybdenum disulfide (MoS_2_)

## Abstract

Due to their atomic-scale thickness, handling and processing of two-dimensional (2D) materials often require multistep techniques whose complexity hampers their large-scale integration in modern device applications. Here we demonstrate that the laser-induced forward transfer (LIFT) method can achieve the one-step, nondestructive printing of the prototypical 2D material MoS_2_. By selecting the optimal LIFT experimental conditions, we were able to transfer arrays of MoS_2_ pixels from a metal donor substrate to a dielectric receiver substrate. A combination of various characterization techniques has confirmed that the transfer of intact MoS_2_ monolayers is not only feasible, but it can also happen without incurring significant defect damage during the process. The successful transfer of MoS_2_ shows the broad potential the LIFT technique has in the emerging field of printed electronics, including printed devices based on 2D materials.

## 1 Introduction

The controlled deposition and patterning of materials is a key challenge in several sectors of technology and, especially, in the rapidly expanding fields of printed electronics [1] and additive manufacturing (AM) [2, 3]. Among the many different techniques which strive for precise, quick, and low-cost AM, the so-called laser-induced forward transfer (LIFT) stands out as a method which is, on one hand, well established for almost three decades [4] and, on the other, particularly appealing for a range of modern applications, such as the printing of functional inks and flexible electronics [5, 6]. The printing of functional inks is an appropriate technique for a variety of applications like photovoltaic systems [7] and the assembly of electronic components [8–10] since it is a wide resolution range technology, suitable for both rigid and flexible substrates, with high speed and, solvent-free manufacturing. LIFT employs short laser pulses to enable the transfer of the specimen of interest from a so-called donor substrate to what is known as a receiver substrate. It is a versatile technique which has facilitated the intact, digitally controlled and large-scale transfer of a diverse class of materials, ranging from biomaterials [11–13] to thin metal films [14, 15].

Ever since the isolation of graphene layers [16], the interest in two-dimensional (2D) and layered materials has grown with a spectacular pace. One especially important class of 2D materials is that of transition metal dichalcogenides (TMDCs) with potential applications in hydrogen evolution reactions (HER) [17, 18], lithium batteries [19], energy storage and conversion [20], flexible electronics [21], optoelectronics [22], and piezoelectric nanodevices [23]. The properties that underlie these applications are exceptional in many aspects, for example the synthesis of TMCDs is facile, they are largely resistant to degradation and they can be tuned between metallic and semiconducting behavior by varying their transition metal atoms, their chalcogenide atoms, or the number of layers. One prominent 2D material of this type of tunability is the prototype TMCD of moldybdenum disulfide (MoS_2_) [24].

Naturally, the need of controlled deposition applies also to 2D materials. The corresponding difficulties and challenges, however, are, in general, more acute than those met in the deposition of bulkier specimens, e.g. thick films of three-dimensional materials or large nanocrystals. Indeed, the atomic-scale thickness of 2D materials makes them more susceptible to damage and their patterning a non-trivial task. These difficulties are reflected to the fact that, with respect to LIFT studies, there have been only a few reports on the transfer of 2D systems. In particular, LIFT [25] and LIBT (laser-induced backward transfer) [26] were used to transfer individual graphene flakes or fragments with [25] or without [26] a so-called dynamic release layer on top of the donor substrate. In a recent work [27], the full potential of LIFT for the digital transfer of graphene was demonstrated for the first time as arrays of graphene pixels were transferred from a Ni donor substrate to a dielectric receiver. Another relevant study reported the transfer of a MoS_2_ monolayer and MoSe_2_ multilayers with a blister-based LIFT technique, but only for individual crystal fragments [28]. Moreover, we should note that laser pulses have been used to synthesize and pattern TMDCs on certain substrates [29], but this so-called laser writing is a different process than laser-induced transfer. Hence, to the best of our knowledge, the laser-induced transfer has not been achieved for any other 2D monolayers (apart from graphene and MoS_2_) and, in particular, the LIFT (or LIBT) printing of arrays of 2D pixels has been reported only for graphene [27].

In this paper we report the first-ever laser-induced transfer of MoS_2_ arrays of pixels, specifically, the transfer of MoS_2_ pixels from a quartz/Ni donor substrate to a SiO_2_/Si receiver. Using the right laser fluence and reduced pressure we succeeded in transferring ultrathin MoS_2_ sheets, as confirmed by a combination of characterization techniques that proved the transfer of intact and high-quality MoS_2_ monolayers with occasional local areas of bi- and tri-layers.

## 2 Experimental details

The synthesis of MoS_2_ was carried out with the chemical vapor deposition (CVD) method. Specifically, MoO_3_ powder was used and sulfur precursor sublimation was applied at 850 °C, with atmospheric pressure under a flow of 30 sccm Ar. Before growth, perylene-3,4,9,10-tetracarboxylic acid tetra potassium salt (PTAS) molecular growth promoters were seeded by spin casting a sub-molecular film uniformly on the sample at 500–2000 rpm and at a concentration of 4 mM in DI water. This process was performed before the deposition of the MoO_3_. Then the MoO_3_ powder is placed in a ceramic boat and the sample with PTAS is mounted on the top of the boat inside the quartz tube. A separate ceramic boat with sulfur powder was placed next to the MoO_3_ powder. The tube was then evacuated to a pressure of 1 mTorr and was subsequently kept at 100 °C for two hours with a flow of 100 sccm Ar. After the tube was firmly dried, the Ar flow was stopped and the temperature was ramped to 850 °C at a rate of 10 °C/min. At the growth temperature, the pressure was adjusted to atmospheric levels by closing the pump valve and flowing Ar to the tube at 30 sccm. Once the atmospheric pressure was established, the sulfur precursor was heated up from 100 °C to 160 °C. After 10 min of growth, the reaction was stopped by reducing the temperature of the sulfur precursor and opening the pump valve. The sample was then cooled at a rate of 10 °C/min [30].

The MoS_2_ deposition on the donor substrate was carried out using a modified surface-energy-assisted process [31]. Specifically, a layer of polycarbonate (PC) was spin-coated (2000 rpm for 60 s) on the as-grown MoS_2_ films and baked in 150 °C on a hot plate for 2 min. A rigid frame made of thermal release tape (TRT, e.g. *Nitto Denko Revalpha©*) was then placed on top of the polycarbonate for handling the PC layer in removing it from the substrate. A water droplet was then dropped around the sample, thus water molecules could penetrate all the way through the MoS_2_ film, resulting in the delamination of the PC-MoS_2_ assembly from the growth substrate. The TRT-PC-MoS_2_ membrane assembly was collected using the TRT frame with tweezers and dried by N_2_ blow. The membrane was then placed on the donor substrate (50 nm Ni on top of a quartz substrate). This was followed by baking the substrate in 150 °C on a hot plate for 2 min and dissolving the PC in chloroform solution for 30 min.

Laser printing experiments were conducted by using a pulsed Nd: YAG (8 ns) laser at 355 nm and a single-shot laser pulse. The experiments were performed at the image plane and used a square mask to create a seventy five-fold (×75) magnification so as to project a 3 mm × 3 mm square into a 40 μm × 40 μm square at the image plane located in the proximity of the focal point of the objective lens (30 mm focal length). During the process, the laser beam is stationary and translation stages in *x*–*y* axes move the substrates (receiver and donor). The spatial increment is 40 μm between the pixels and the temporal increment is 0.1 s.

The experiment was conducted under reduced pressures ranging from 40 mbar down to 0.9 mbar, with the optimal transfer achieved at 0.9 mbar.

**Figure 1:**
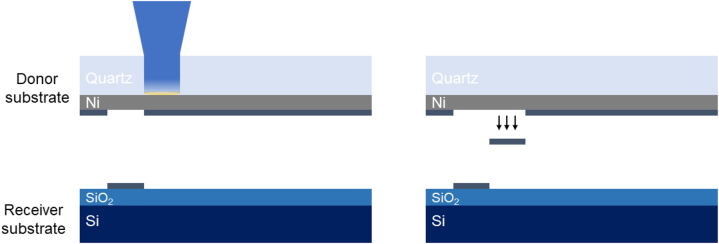
Laser induced forward transfer (LIFT) process. The laser beam propagates through the donor substrate and induce the transfer of the MoS_2_ pixel.

Figure 1 depicts the process as it was carried out for the specific experiment. Specifically, in the Laser-Induced Forward Transfer (LIFT) experiment a pulsed laser is used to transfer the material of interest from a so-called donor substrate to a receiver substrate. A typical donor substrate consists of a transparent layer (e.g. quartz) and an assistant layer, known as a dynamic release layer (DRL). The material to be printed (e.g. MoS_2_) with LIFT is either grown or transferred on the DRL (here Ni). Laser pulses then propagate through the transparent donor substrate and are absorbed by the DRL. Eventually, and if the incident laser energy fluence is above a certain threshold value, the overlayer material is ejected from the donor and is propelled towards the receiver substrate.

For the purposes of the experiment the donor and the receiver substrate were in contact.

Micro-Raman spectra were recorded with a Renishaw inVia Reflex microscope operating at 514 nm using a 100× objective lens with a 0.7 μm laser spot diameter on the sample. The laser power was kept at 0.3 mW to avoid heating the material during the measurements. Raman spectra were collected within the 30–100 cm^−1^ region and the acquisition time was 60 s with three accumulation rounds for each spectrum. Scanning electron microscope (SEM) measurements were performed using the ultra-high resolution JEOL JSM-7401f field emission scanning electron microscope (FESEM), with an accelerating voltage up to 2 kV. The samples have been characterized without any prior metallization step. Atomic Force Microscopy (AFM) measurements have been performed using the Veeco diInnova 840-012-711 operating at tapping mode.

## 3 Results and discussion

Printing large patches of 2D materials with the use of a laser requires the optimization of multiple parameters such as the laser fluence, the proper adhesion to the donor and the receiver substrates, the size and shape of the laser beam spot, the distance that separates these two substrates and the pressure in the region between them. The identification of the optimal set of these parameters is a nontrivial task, especially when there is hardly any prior knowledge for the transfer of a particular system. This note applies definitely to MoS_2_, for which, as we stressed above, there has been only one previous transfer [28] of just individual MoS_2_ crystal fragments and no study on printing larger structures with spatial order and periodicity.

For the laser printing experiments one has to choose a donor substrate which is capable of absorbing the laser light, which, in our case has a wavelength of 355 nm. For this matter, the MoS_2_ layers were initially deposited on a Ni thin film (thickness of 50 nm) which itself was on top of a thicker quartz film (thickness of 1 mm).

One the other hand, the receiver substrate in this study was a Si single crystal wafer with 200 μm thickness and with an oxide of 300 nm thickness on top. Such substrates (Si/SiO_2_) with this oxide thickness make the optical detection of single-layer MoS_2_ possible and also have good adhesion with MoS_2_ [31–33]. The particular oxide thickness has been established by the correlation between contrast and thickness as measured by atomic force microscopy (AFM) [34].

**Figure 2:**
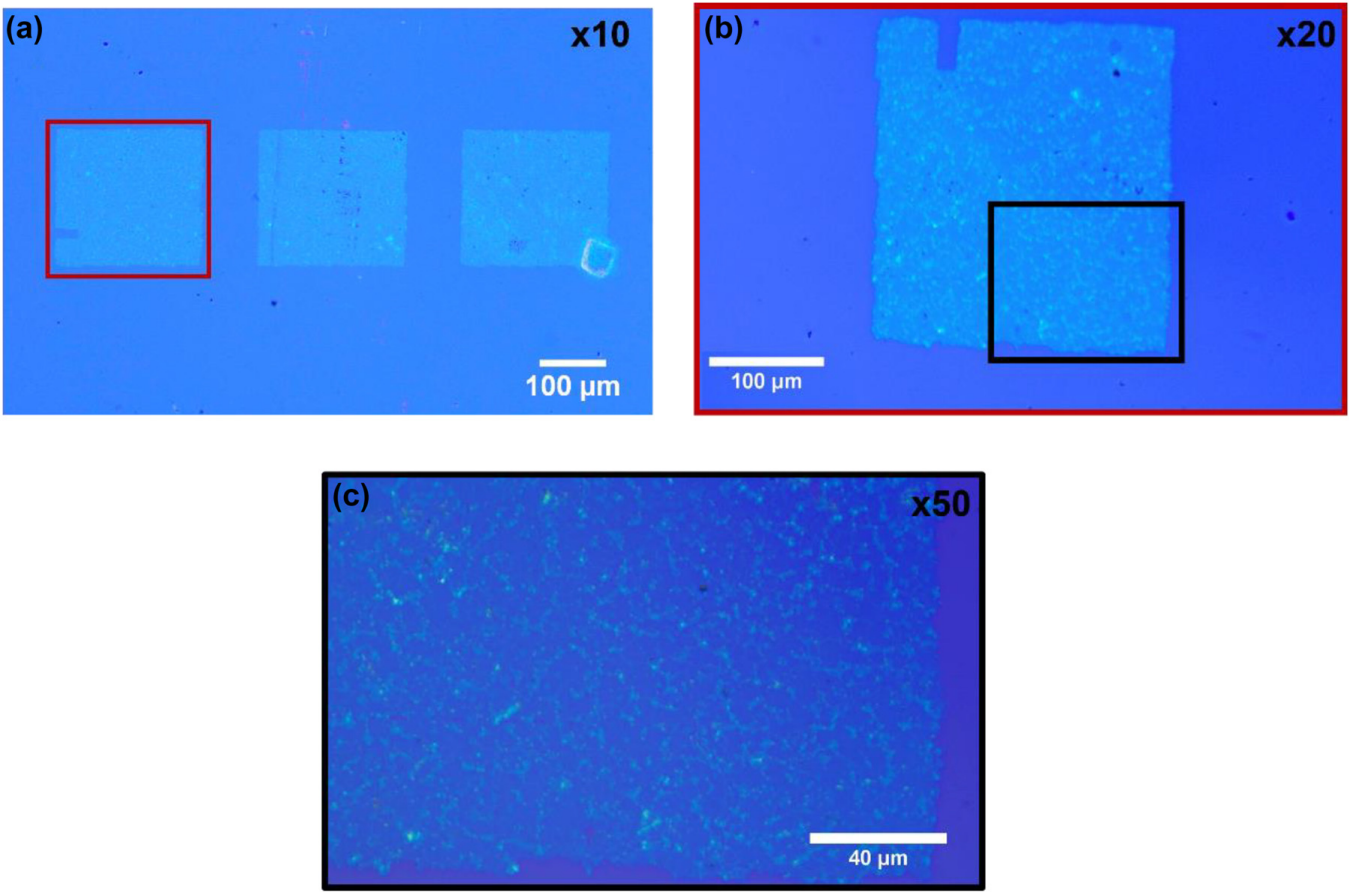
Optical microscope images of arrays of MoS_2_ pixels printed on a SiO_2_/Si receiver for different magnification levels: (a) ×10, three different arrays of pixel are depicted and each array comprises a grid of 6 × 6 pixels, b) ×20 zoomed in view of the leftmost array shown in (a), and (c) ×50 magnification of the black rectangle of (b).

**Figure 3:**
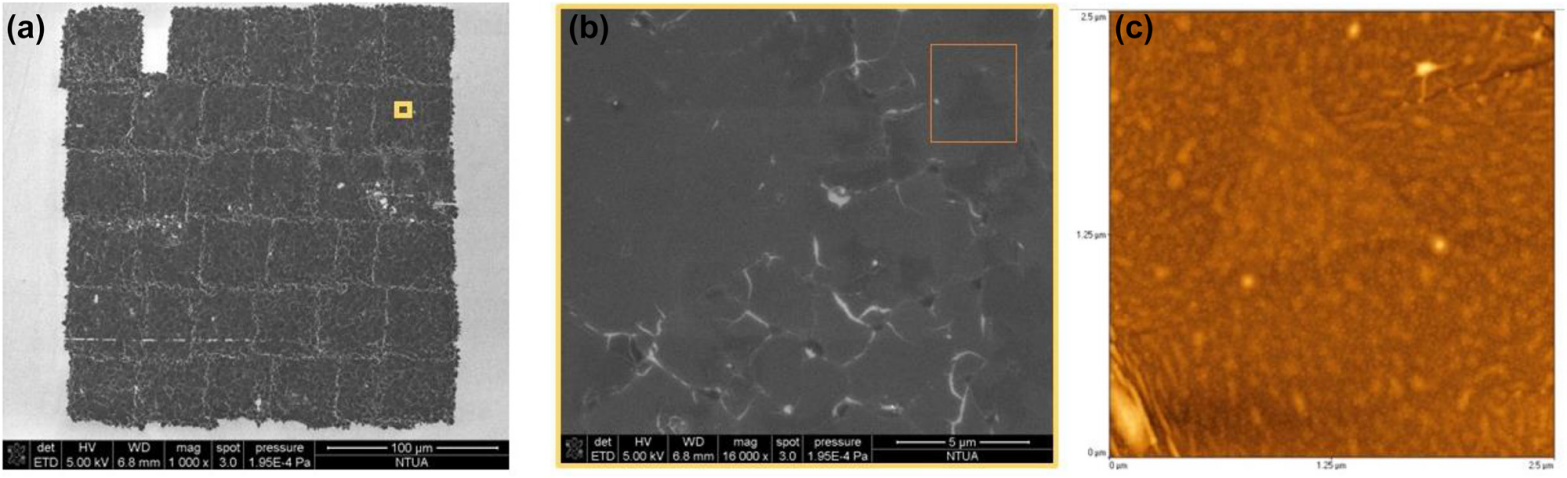
SEM images of LIFT-printed MoS_2_ arrays with (a) a ×1000 magnification, and (b) ×18000 zoomed-in view of the yellow region of (a). SEM images present a well-defined transfer of pixels. (c) AFM image of a region 2.5 μm × 2.5 μm, which corresponds to orange rectangle shown in (b) and which depicts an intact printed MoS_2_ single layer with a triangle.

The applied laser energy density (*E*_*i*_) is an important factor that decides whether the transfer will take place and the overall quality of printed specimens. We have found that pixels with good quality of MoS_2_ transfer occurred for values of *E*_*i*,_ at 80 ± 5 mJ/cm^2^. On the other hand, for *E*_*i*_ values above 100 ± 5 mJ/cm^2^ the MoS_2_ transfer was not of good quality, either due to the ablation of the receiver substrate ablation or due to the contamination of the MoS_2_ printed sheets with nickel. Hence, the results we report in the following were obtained with the lower *E*_*i*_ value of 80 mJ/cm^2^. In addition to the *E*_*i*_ parameter, and based also on the experience with the LIFT experiments on graphene [27], the pressure in the region between the donor and the receiver substrates is another critical factor. Assuming that the thickness on the donor material is uniform, then to achieve a uniform printed layer a key parameter is the laser beam uniformity. Top hat laser beam profiles result in uniform printed thickness of MoS2. By selecting a reduced pressure of 0.9 mbar and a top hat laser beam profile, we were able to print MoS_2_ pixels and arrays with uniform and continuous coverage of the receiver substrate, as shown in Figures 2 and 3. Specifically, Figure 2(a) depicts a 6 × 6 array of MoS_2_ pixels printed on the SiO_2_/Si receiver substrate. The good optical contrast between the arrays and the receiver substrate reveals that the former has well-defined, sharp edges. As shown in Figure 3, each array comprises individual MoS_2_ pixels whose size is approximately 40 μm × 40 μm. The pixels are sequentially transferred by directing the laser to neighboring positions on the donor substrate. In this way, they form a continuous printed film on the receiver side with a total area of approximately 240 μm × 240 μm.

The array shown in Figure 2(b) was characterized further with SEM. In particular, Figure 3 presents two SEM images for the printed array and for two different magnifications. Figure 3(a) clearly shows that the printed film is a 6 × 6 array of pixels. The edges of the pixels are clear and have white boundaries and as shown in Figure 3(b), such white lines are present also inside the pixels. In addition, there are some darker areas inside the pixel shown in Figure 3(b). As discussed below, AFM characterization of the latter dark areas revealed that they relate to the existence of smaller MoS_2_ triangles underneath the printed MoS_2_ monolayer film. Finally, we note that one pixel is actually missing on the last row because the laser did not fire at this spot. Differences in the shape and the area of the pixels are related to the fact that the firing of the laser occasionally does not happen exactly at the same point as the movement of the motorized stages.

The darker area marked within the orange box (whose dimensions are approximately 2.5 μm × 2.5 μm) on the SEM image of Figure 3(b) corresponds to a single-layer MoS_2_ area where a triangle has been transferred intact with LIFT. Indeed, more detailed characterization of this area with AFM (Figure 3(c)) show the characteristic triangular shape of a MoS_2_ flake.

**Figure 4:**
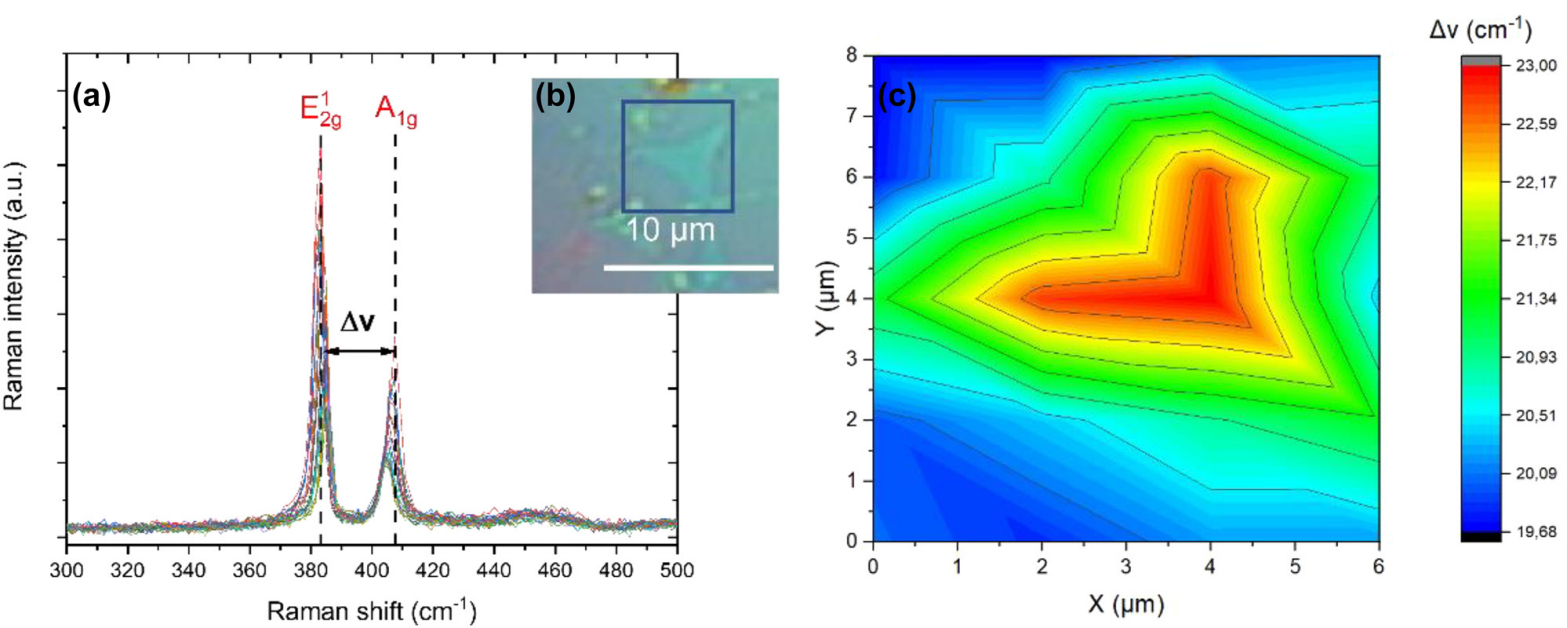
Characterization of an area of the printed MoS_2_ layer with Raman spectroscopy. The image in the middle is from an optical microscope with ×100 magnification and shows the characterized region. The Raman graph on the left depicts 30 different Raman spectra which correspond to 30 points within the marked area on the inset (b) in the middle. The image on the right is a Raman colour map and shows the range of the μv within the blue box of (b) with an area of 6 μm × 8 μm.

**Figure 5:**
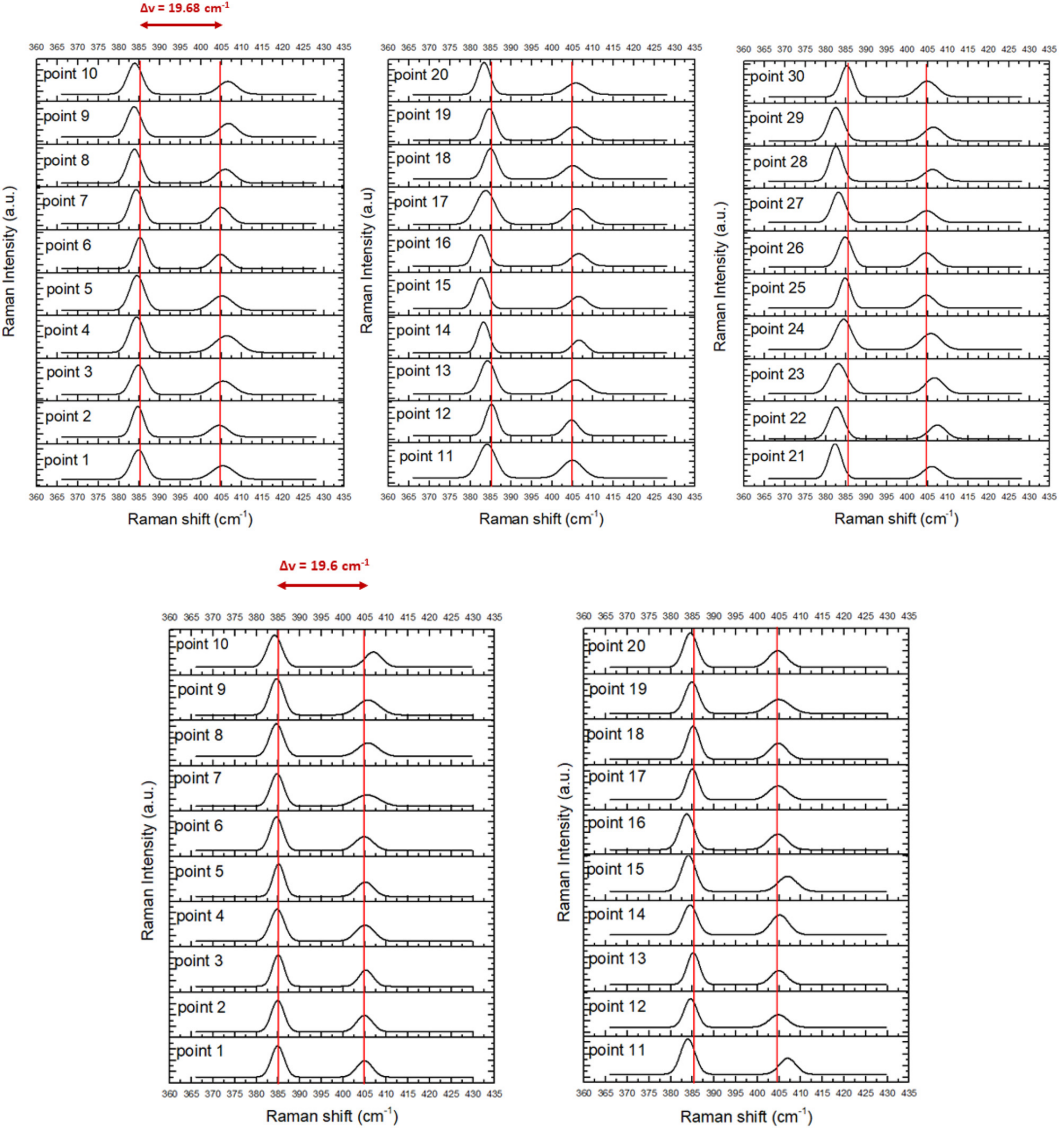
Raman spectrum of mapping measurements in two different regions of the sample with a) 30 points and corresponds to the region of Figure 4 and b) 20 points of another region on the sample.

**Figure 6:**
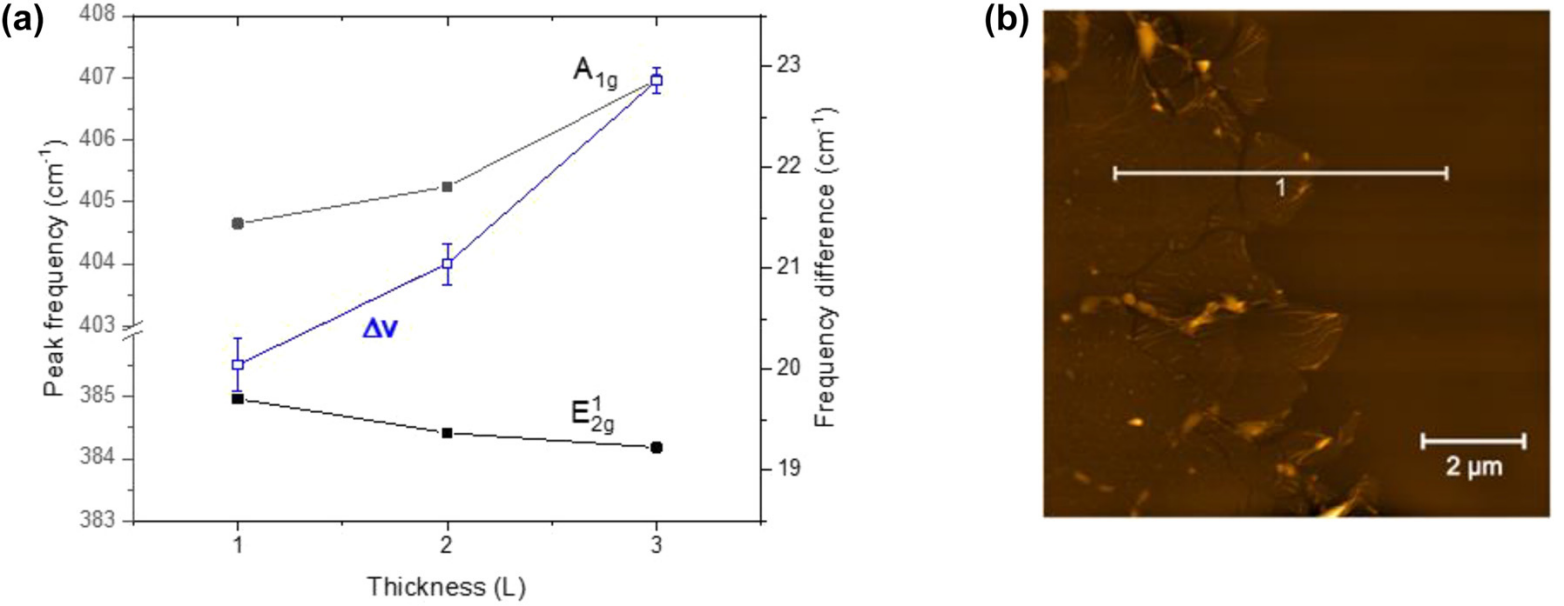
(a) Dependence of the peak frequencies of the E_2g_^1^ and A_1g_ modes of the area shown in Figure 3(b) on the number of MoS_2_ layers. (b) AFM image of the edge of the transferred array

To gain more information on the structural details of the printed MoS_2_ films and their overall quality we carried out Raman spectroscopy measurements. In particular, we selected a region within the area shown in Figure 2(b) and obtained the corresponding Raman data which are presented in Figure 4. As shown in the inset of Figure 4(b), optical microscopy reveals that this area has distinct MoS_2_ triangles. For the 6 μm × 8 μm region inside the blue box of Figure 4(b), we accumulated data from 30 different points, which were uniformly distributed within the box. Figures 4(a) and 5(a) show the Raman spectra for these points and for the two characteristic MoS_2_ vibrational modes E_2g_^1^ and A_1g_. Figure 5(b) presents the Raman spectra for another sample region. The measured spectra of Figures 4 and 5 are in agreement and overall give a reliable indication that the transferred MoS_2_ is consistent with the phase 2H [35]. The in-plane E_2g_^1^ mode results from the out of phase vibration of two S atoms with respect to their bonded Mo atom, while the A_1g_ mode is associated with the out-of-plane vibration of only S atoms in opposite directions [36]. The Raman spectra of Figures 4(a) and 5 provide evidence for the quality and the number of layers of the LIFT transferred MoS_2_. According to previous thickness-dependence Raman studies [37, 38], on TMDCs, the distance (Δ*ν*) of the main bands (E_2g_^1^, A_1g_) is an indication of the number of MoS_2_ layers. In Figure 4(c) we used the Raman spectra of Figure 4(a) to form a color map based on the distances (Δ*ν*) between the two main bands (E_2g_^1^, A_1g_) so as to explain the distribution of the layers inside the blue marked region of the transferred MoS_2_. As can be seen in the pertinent color map of Figure 4(c), this area contains two MoS_2_ triangles vertically grown on the top of the monolayer MoS_2_. Specifically, Δ*ν* values with a range from 19.6 cm^−1^ to 20.51 cm^−1^ indicate the existence of a monolayer (1L) and its region (which surrounds the triangles in this case) is marked with blue in the figure. Likewise, Δ*ν* values from 20.93 cm^−1^ to 21.90 cm^−1^ indicate a bilayer (green) and from 22.17 cm^−1^ to 23 cm^−1^ three layers (red). Importantly, there is no dark line or region found in the Raman mapping image, which would indicate that this region cannot be recognized by Raman due to the lack of the modes E_2g_^1^ and A_1g_. In other words, the Raman color map confirms the continuous nature of the printed film. Furthermore, from the Raman spectra we found that the full width at half maximum (FWHM) of the E_2g_^1^ mode remains approximately constant and equal to a small value of 4.9 ± 0.5 cm^−1^. Additionally, we measured a double resonance 2LA(M) at around 460 cm^−1^ with broad and small amplitude. The above two findings indicate good crystalline quality and that the transferred pixels were not affected by the laser irradiation conditions. The fact that this transferred array pertains to a monolayer was also confirmed by AFM measurements which obtained a thickness of 0.89 ± 0.3 nm (see Figure 6b) [39] which is in alignment with the thickness of MoS_2_ on the donor. It is noted and in other studies [36, 37] the height of a monolayer MoS_2_ on bare substrates like SiO_2_ is about 0.8–1.0 nm.

For single layer, bi-layer and tri-layer MoS_2_ the Raman spectra we studied in Figures 4 and 5 show strong signals from both the in-plane E_2g_^1^ and the out-of-plane A_1g_ vibration. An overall graph (Figure 6a) sums up the positions of the A_1g_ and E_2g_^1^ modes and their relationship with the number of layers in correlation to their spectral distance Δν. The behavior as a function of film thickness has several intriguing characteristics. Specifically, we find (Figure 5a) that the E_2g_^1^ vibration softens (red shifts), while the A_1g_ vibration stiffens (blue shifts) with increasing sample thickness, in agreement with previous studies [36, 37, 40].

## 4 Conclusions

In conclusion, by finding the optimal set of LIFT parameters (e.g. laser fluence, pressure, distance between donor, and receiver substrates) we achieved for the first time the transfer of MoS_2_ arrays of pixels from a quartz/Ni donor to a SiO_2_/Si receiver. The good quality of the printed film was verified by various characterization techniques, namely optical microscopy, scanning electron microscopy, Raman spectroscopy, and atomic force microscopy.

The results obtained in this study have important implications for the field of printed electronics as they clearly show that LIFT is a non-destructive process which is capable to transfer even delicate 2D materials without triggering extensive defect damage or degrading their quality in terms of crystallinity. Hence, our findings suggest that, in the near future, LIFT technology can be expected to controllably transfer also other 2D materials with a quality comparable to other technologies [41]. Such a breakthrough can lead to the larger-scale fabrication of low-cost, one-step, printed electronics based on 2D or layered materials.
